# Measuring the Impacts of Community-based Grasslands Management in Mongolia's Gobi

**DOI:** 10.1371/journal.pone.0030991

**Published:** 2012-02-01

**Authors:** Craig Leisher, Sebastiaan Hess, Timothy M. Boucher, Pieter van Beukering, M. Sanjayan

**Affiliations:** 1 Central Science, The Nature Conservancy, Arlington, Virginia, United States of America; 2 Hess Environmental Economic Analyst, Haarlem, The Netherlands; 3 Department of Environmental Economics, Institute for Environmental Studies, VU University, Amsterdam, The Netherlands; DOE Pacific Northwest National Laboratory, United States of America

## Abstract

We assessed a donor-funded grassland management project designed to create both conservation and livelihood benefits in the rangelands of Mongolia's Gobi desert. The project ran from 1995 to 2006, and we used remote sensing Normalized Differential Vegetation Index data from 1982 to 2009 to compare project grazing sites to matched control sites before and after the project's implementation. We found that the productivity of project grazing sites was on average within 1% of control sites for the 20 years before the project but generated 11% more biomass on average than the control areas from 2000 to 2009. To better understand the benefits of the improved grasslands to local people, we conducted 280 household interviews, 8 focus group discussions, and 31 key informant interviews across 6 districts. We found a 12% greater median annual income as well as a range of other socioeconomic benefits for project households compared to control households in the same areas. Overall, the project generated measurable benefits to both nature and people. The key factors underlying project achievements that may be replicable by other conservation projects include the community-driven approach of the project, knowledge exchanges within and between communities inside and outside the country, a project-supported local community organizer in each district, and strong community leadership.

## Introduction

Around the globe, grasslands provide livelihoods for nearly 800 million people and are a crucial source of livestock forage and wildlife habitat [1]. However, three quarters of the world's grazing lands are so degraded that they have lost more than 25% of their capacity to support animals [2]. Most of the world's grasslands are found in temperate regions, and these temperate grasslands have the distinction of being the most altered terrestrial ecosystem on the planet [3]. In traditional pastoral systems, grasslands and pastoral communities are mutually dependent, as grazing is often necessary to maintain historic plant community structure. Experiments have shown that temperate grasslands which are grazed tend to support greater biodiversity and plant biomass than ungrazed areas, and diversity and primary productivity are often linked [4]–[11]. Therefore, improved grassland management has the potential to improve both grassland health and rural livelihoods. In this study, we assess the impacts of a community-based grassland conservation project in Mongolia's Gobi desert on both conservation and livelihood goals, and identify factors that contributed to the project's achievements.

### Grassland degradation in Mongolia

The Mongolian grasslands have been home to herders for thousands of years. However, recent decades have seen a significant increase in grazing pressure. When Mongolia began transitioning to a market economy in 1990, Soviet-era subsidies came to an end, rural production collectives disintegrated, and socioeconomic conditions deteriorated. Many who lost their jobs after 1990 took up subsistence herding, and the number of herding households doubled between 1990 and 1998 [12]. Many of these new herders were inexperienced at pasture and livestock management. Moreover, the services once provided by the livestock management collectives, such as coordination and transport for seasonal moves, the upkeep of water sources, and veterinary services, ended with the dismantling of the collectives. This led to a reduction in livestock mobility, which resulted in the overgrazing of pastures around district and provincial centers and around water sources. Despite this, there was a large increase in the number of animals due to the generally favorable weather conditions during the initial years after the end of socialism. In 1990, Mongolia had 26 million domesticated animals. In 1998, it had 33 million, an increase of 27% [12]. Though an estimated 10 million animals died due to harsh winters between 1999 and 2002, livestock numbers increased again after that time, reaching 44 million by the end of 2009 [13]. However, from late 2009 to early 2010, Mongolia was hit once more by an especially harsh winter, exacerbated by a drought the previous summer, and an estimated 8 million animals died (Ministry of Food, Agriculture and Light Industry).

In 1995, to address national park management and the sustainability of herders' livelihoods, the German government began funding a joint project with the Mongolian government on the Conservation and Sustainable Management of Natural Resources which focused, *inter alia*, on creating and training buffer zone management councils around Gobi Gurvan Saikhan National Park. In 1998, a second phase of the project created Community Organizations to improve pasture management, develop alternative livelihoods, and strengthen cooperation among local communities, the park administration, and district governments. Improved pasture management included coordinating the moves on and off pastures for all participating herders, improving water sources for livestock, and developing specific winter grazing areas for Community Organization members. The development of Community Organizations was supported by locally hired community organizers who were part of the project staff. There was one community organizer in each district, and their role was to organize and encourage the communities and act as a liaison with local government, resource agencies, and the rest of the project team. The project ran for another eight years, comprised 12 districts across 3 provinces, and covered 13.5 million hectares. When funding support to the project ended in 2006, 83 Community Organizations had emerged, involving 1,175 households, or about 14% of the households in the project area.

The project area is in the arid lands of the Gobi, a region characterized by low levels of rainfall with high variability both spatially and temporally [14], which results in a non-equilibrium ecological system [15]. Over the last decade, precipitation in the project area averaged 126 mm per year, with an inter-annum variation of 39% [16]. The elevation of the region ranges from 706 m to 2,825 m. In the project area, three pasture types can be distinguished: dry, shrubby saxaul pastures (*Haloxylon ammodendron*) for camel grazing; grasslands pasture for small livestock; and mountain pasture for horses and yak [17]. Pasture management techniques under the project where based on the traditional approach of the entire community moving livestock to areas with recent rainfall and setting aside area for winter grazing.

In 2009, approximately 33,500 people lived in the project area [13]. Almost all of them depended on livestock herding as their principle livelihood. In the project areas, goats make up approximately 65% of the livestock followed by sheep (25%), horses (5%), camels (3%) and cows (1%). Government income poverty rates for the 12 project districts vary from 52% to 26% with an average of 36% [13]. The project area is ethnically homogeneous, and literacy in the study area is high (98%).

## Methods

To measure the project impacts, the study team conducted ecological and socioeconomic assessments across the area covered by the project, four years after its conclusion. Field work took place in June and July 2010, with 10 days for the ecological assessment and 38 days for the socioeconomic assessment.

**Figure 1:**
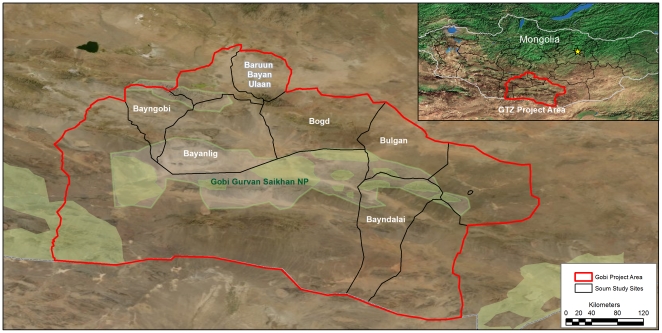
Map of the Gobi project area and study sites. Shows the names of the 6 districts where the study was conducted and the national park.

The study team sampled six project districts (Figure 1). These districts were selected based on a cross-section of saxaul, grassland and mountain pastures and the number of active Community Organizations.

### Ecological assessment

For the ecological assessment, a remote sensing approach complemented by ground-calibration was used. Using time-series Normalized Differential Vegetation Index (NDVI) observations, one can examine the dynamics of a growing season [18]–[20] and anthropogenic changes such as overgrazing [21]–[23]. To compare NDVI before and after the conservation initiative in both project and control sites [24], we used Advance Very High Resolution Radiometer (AVHRR) (8 km×8 km pixels) and Moderate Resolution Imaging Spectroradiometer (MODIS) imagery (250 m×250 m pixels). The AVHRR data were analyzed by pre- and post-conservation intervention timeframes. Both AVHRR and MODIS data were used to compare community-organization grazing sites (“treated”) and non-community organization grazing sites (“control”). MODIS NDVI data are not directly comparable to AVHRR NDVI data, so the data were analyzed separately and the averages combined (Table 1).

**Table 1:** Analytical structure of the ecological assessment.

Comparison	Imagery Source	Unit of Analysis	Scope
Before versus after project	AVHRR	By year, pre-conservation (1982-99) versus post-conservation (2000-06)	6 of the 12 project districts
Treated versus control sites	MODIS	By year (2000-2009)	39 treated and 37 control data points
Treated versus control sites	AVHRR	By year (2000-06)	18 treated and 18 control data points

**Figure 2:**
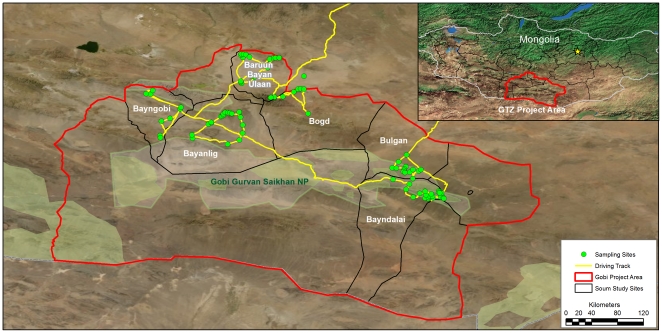
Ecological assessment sampling sites. Shows the 3,200 km driving track for the ecological assessment team and the 76 sampling sites where ground calibrations were conducted.

Ground calibrations from the center of all the grazing sites sampled were collected in the field (Figure 2). The treated sample sites were selected based on the following criteria: (i) a grazing area managed by a Community Organization created by the project; (ii) in the middle range of elevation for the grazing area type; (iii) an area without trees at least 3 km×3 km; and (iv) the same vegetation type as the surrounding area.

The control sites were matched with the treated sites based on the following criteria: (i) within 20 km of the treated pasture; (ii) same habitat type; (iii) elevation ±100 m; and (iii) same rainfall as per NASA Monthly Global Precipitation data.

### Socioeconomic assessment

For the socioeconomic assessment, we used the World Bank's definition of poverty which comprises three elements: opportunity, empowerment and security [25]. To make the definition of poverty measurable, the three elements of poverty were subdivided into 13 focal areas (Table 2). Each focal area was assessed using the qualitative and quantitative tools of key informant interviews, focus group discussions, and a structured random survey of households as detailed below. The qualitative tools are predominantly used for the interpretation of quantitative results.

**Table 2:** Poverty focal areas.

Opportunities	Empowerment	Security
Income	Governance mechanisms	Health
Alternative livelihoods	Community participation	Social cohesion
Livestock management	Benefits to women	
Pasture management		
Access to credit		
Housing		
Durable goods		
Education		

The qualitative analysis consisted of semi-structured key informant interviews with government officials, community organizers, and community leaders as well as focus group discussions with members of project Community Organizations. The focus group discussions followed a written protocol and included women-only discussions. A trained local facilitator guided the discussions. Interviews and focus group discussions covered each of the 13 poverty focal areas.

A household survey provided the data for the quantitative analysis. The questionnaire for the survey was developed with the help of local experts and finalized after two rounds of local pre-testing. Topics covered in the survey were based on the same 13 poverty focal areas (Text S1). The household survey enumerators consisted of experienced Mongolian nationals some of whom knew the study area.

A sample frame for the survey was drawn up by collecting population data at district and sub-district levels, in cooperation with local government officials and former community organizers. Eligible project households were members of active Community Organizations and had to already have been herding in the district before 2002 when project activities ramped up.

Control households were selected from the same districts as the project households. This ensured that the treated and control households had faced similar weather conditions. Weather can differ significantly on a local scale and is the main determinant for grazing conditions and hence herders' livelihoods. This approach also controlled for a number of other potentially confounding variables, such as distance to markets, access to government services, and the presence of development projects in the community. To limit the possible influence of project households' behavior and pasture management practices on the control group, households that had winter camps and pastures close to those of the Community Organization households were excluded from the sample frame.

A random sample was drawn from the treated household sample frame. A matching stratified random sample was then drawn from the control sample frame. The stratification for the control sampling was based on the distribution of household welfare in 2002 in the treated sample. For this the official government welfare grouping indicator was used: very poor = 0–50 animals; poor = 51–100 animals; average = 101–500 animals; better-off = 501–1000 animals; and wealthy >1000 animals. While this indicator is only a rough proxy for welfare, it was the sole indicator available and was therefore used to ensure that pre-intervention both the treated and control households were similarly “well-off”. Goats and sheep accounted for 90% of the total livestock in the project area.

The data were analyzed using SPSS 15.0 and included propositional comparisons and statistical significance using T-tests, Mann-Whitney U, and Chi^2^ (Data set S1).

### Ethics Statement

We obtained verbal consent from participants before conducting household surveys. During verbal consent, participants were informed about the survey, its purpose, and how the data would be utilized. This project was administered by The Nature Conservancy, which does not have a formal Institutional Review Board, but the assessment plan was reviewed and approved by the senior level of the organization. Verbal permission for the research was granted by each of the district-level governments where the survey was conducted. To avoid confidentially issues, names and addresses of respondents were excluded in the data analyses.

## Results

### Ecological assessment

**Figure 3:**
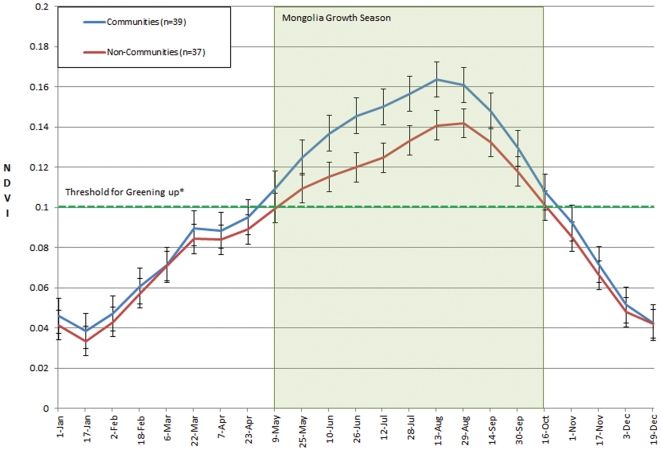
Ten-Year Seasonal Growth Curve Comparison (NDVI). Shows how the 10-year average growth of the 39 community pastures compares with the average from the 37 non-community matched control sites (t-test = 2.715, df = 18, *p* = 0.014). The community pastures had a longer growing season and higher peak NDVI and produced more biomass than the non-community control pastures. *Above 0.1 is when plants can be grazed [26]. Error bars = one standard error.

Comparing the average seasonal plant growth curve from 2000–2009, a clear difference between the community-managed areas and non-community areas can be seen (Figure 3). Based on a threshold of 0.1 NDVI for when vegetation becomes available for grazing [26], the season was longer in the community-managed areas than non-community sites (∼180 days vs. ∼160 days) (t-test = 2.715, df = 18, *p* = 0.014). The green-up of Community Organization sites occurred earlier and more rapidly in the spring by almost two weeks, allowing for livestock to recover more quickly from the winter.

**Figure 4:**
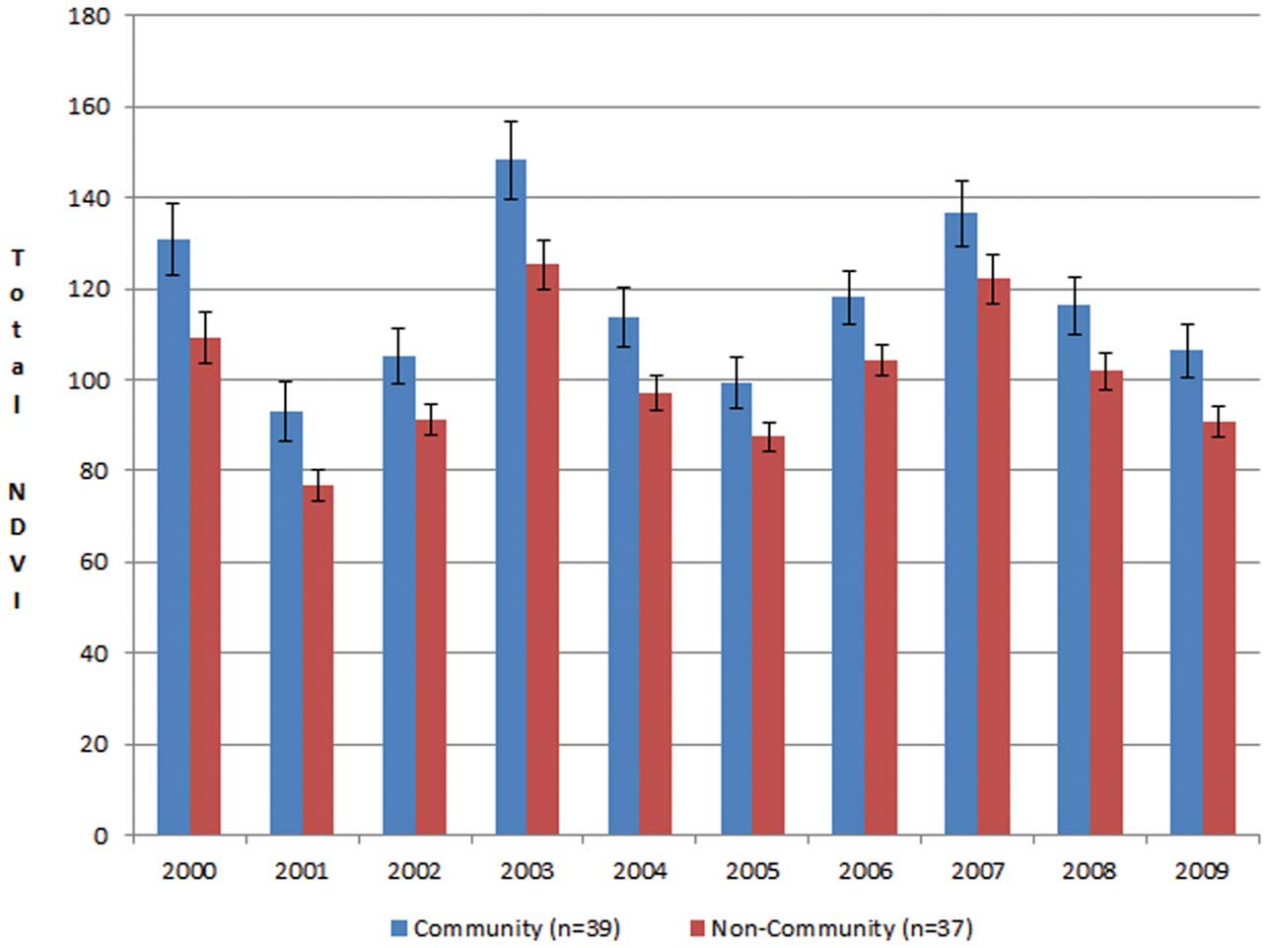
Seasonal Biomass Growth (TI-NDVI). Shows how the 39 community pastures' seasonal growth in biomass compares to the 37 non-community controls (t-test = 2.103, df = 18, *p* = 0.05). Variation year-to-year is due to rainfall. Error bars = one standard error.

The peak growth of grass in the community-managed areas was 14.8% greater (t-test = −2.039, df = 74, *p* = 0.045). This means that plant growth was denser, and there was more forage available for livestock and wildlife. On average, the overall green season ten-year NDVI for community sites was 15.4% greater than in non-community sites (t-test = 2.715, df = 18, *p* = 0.014). In addition, Time-Integrated NDVI (TI-NDVI) data, which estimates the accumulated yearly plant growth on a site, indicated that, on average, community sites had 15.2% more plant biomass than non-community sites from 2000–2009 (t-test = 2.103, df = 18, *p* = 0.05) (Figure 4).

To test whether Community Organization sites had historically higher levels of plant growth, we compared the sample and control sites pre- and post-conservation intervention using TI-NDVI data from 1982 to 2006. The difference between yearly growth-season TI-NDVI between community and non-community control sites was less than 0.4% for the period 1982 to 1999 (pre-conservation). For the period 2000 to 2006 (after phase 2 of the project started), the community sites had a 6% higher TI-NDVI than control sites.

Pasture management can be most important during drought years, and therefore we analyzed whether Community Organization sites were specifically better off during drought years (those in which the annual TI-NDVI was below the three decadal average for all the sites). During the two decades before the conservation intervention, both the community and non-community areas had almost the same TI-NDVI during drought years (within 0.02%). However, after the project was initiated, the community sites had a 14% higher TI-NDVI in the ten drought years between 1982 and 2006 of 1984–86, 1991,1995, 1996, 2001, 2002, 2004, 2005 (t-test = 1.951, df = 6, *p* = 0.099).

### Socioeconomic assessment

A total of 280 households were sampled, made up of 154 members of active Community Organizations and 126 non-member households. Females comprised 44% of the sample. Demographically, the average household interviewed consisted of 4.3 members and had 2.7 children, which is similar to national averages. Twelve percent of the sample was female-headed households. The average age of the respondent was 46 years with a range of 17 to 83.

Thirty-one semi-structured key informant interviews were conducted, as well as eight focus group discussions with members of 13 project Community Organizations (three women-only, two men-only, and three mixed-gender discussions).

The survey data showed households that were members of Community Organizations to be significantly better-off, based on a number of metrics (Table 3), and participants in the study were generally positive about the project.

**Table 3:** Summary of quantitative analysis results.

Poverty focal area	Difference between member and non-member households	*p*- value	Statistical test
Income	Member median annual income 12% higher	p = 0.1	Mann-Whitney U = 8595; n = 278
Alternative livelihoods	2.4% vs. 0.7% of total income and at least 13 new income activities	p<0.001	Mann-Whitney U = 7479; n = 278
Livestock (management)	No statistical difference in herd size	p = 0.6	t-test; df = 278
Pasture management I	17% more members have access to winter pastures	p = 0.002	Chi2 = 9.533; df = 1; n = 280
Pasture management II	12% more members have the ability to produce or buy hay & fodder	p = 0.043	Chi2 = 4.086; df = 1; n = 280
Access to credit	18% more members have loans	p = 0.001	Chi2 = 10.555; df = 1; n = 280
Housing	No statistical difference in the number of *gers*	p = 0.5	Mann-Whitney U = 9202; n = 279
Durable goods I	10% more members own a television and satellite dish	p = 0.085	Chi2 = 2.957; df = 1; n = 279
Durable goods II	11% more members own a truck or car	p = 0.059	Chi2 = 3.576; df = 1; n = 279
Education	16% more member households have someone attending university or with a university education	p = 0.001	Chi2 = 11.955; df = 1; n = 276
Governance mechanisms	31% more members feel the relationship with the park administration has improved	p<0.001	Chi2 = 19.369; df = 2; n = 280
Community participation	No statistical difference in perceived influence on local government	p = 0.7	Chi2 = 0.182; df = 1; n = 276
Benefits to women	25% more members see an improvement in the role of women	p<0.001	Chi2 = 17.593; df = 2; n = 270
Health	No statistical difference in access to health care	p = 0.5	Chi2 = 2.286; df = 3; n = 280
Social cohesion	No statistical difference in the occurrence of disputes	p = 0.3	Chi2 = 4.082; df = 3; n = 280

Economically, member households were better-off. Survey data show that average annual income of community member households is considerably higher than that of non-member households at MNT 4,281,688 and MNT 3,379,090 respectively (USD 3,244 and USD 2,560). However, two households with income level of over MNT 20,000,000 (USD 15,100) skewed this average. When those outliers are removed, the result is an 18% greater average income for project households than non-member households (MNT 3,977,855 versus 3,379,090 respectively). The median annual income was also higher for project households by 12% than for non-member households.

In almost all focus group discussions, participants identified increases in income as an important impact of the project and the Community Organizations. On average, selling cashmere was the most important income source for all households, followed by the income from selling live animals for Community Organization households and government welfare payment for non-member households. Member households derive significantly greater income from selling live animals, alternative income sources, and selling processed animal products. A little over a fifth (22%) of the difference in income between member and non-member households is attributable to income derived from selling value-added items and providing tourism services initiated after project-run training sessions. Average income from alternative income sources (making handicrafts, growing vegetables, and providing tourism services) differed by MNT 77,470 (*p*<0.001; Mann Whitney U = 7492; n = 278) and average income from selling processed animal products differed by MNT 55,012 (*p* = 0.01; Mann Whitney U = 8459; n = 278). The income difference between member and non-member households from selling live animals is MNT 411,028 *(p* = 0.004; Mann Whitney U = 7788; n = 278).The items sold include felt products, dairy products (milk, yoghurt, cheese, ice-cream, sweet cream), souvenirs, cookies and other pastry/baking products, sausages, boots, belts and other leather products, yarn and other wool products, fresh and preserved vegetables, furniture, medicinal teas, building blocks from cement, and fuel briquettes. Tourism services include food, accommodations and acting as guides to the area.

The Gobi project also improved member households' access to credit. Buffer Zone Councils set up around the park provided, *inter alia*, microcredit using capital supplied by the local government and the project. Community Organizations also set up community funds from which credit was provided to members. Of the 13 Community Organizations that participated in the focus group discussions, only two still had a functioning community fund that was actively reporting to community members in 2010. Several key informants mentioned that the community funds had been emptied to buy hay and fodder during last year's hard winter. In some districts, the Buffer Zone Councils were criticized for not providing credit anymore.

In addition, a greater percentage of member households own a television and satellite dish, and a car or truck.

A good winter pasture or a source of winter fodder is crucial for livestock survival in a harsh winter, and 42% of member households have the opportunity to reserve a winter pasture area compared to 25% of non-member households. A greater proportion of member households (25%) also have the ability to produce or buy hay and fodder for the winter than non-member households (13%). In both cases, the Community Organizations acted as the focal point for organizing these resources.

Member households also see benefits in governance, education and empowerment. The Community Organizations are a legally recognized rural civil society organization within the civil code. This status and a provision in the land law allowed contracts on communal land management to be made between local government and the Community Organizations and gave the organizations rights to manage designated pastures and undertake conservation activities. The organizations also fostered better relationships with the national park administration. Even though focus group discussions frequently noted that the relationships with the national park administration has declined since the project ended, 31% of member households still viewed the relationship as improved compared to before the project, while only 6% of non-member households held the same view.

A greater proportion of community households have a member in university or with a university degree (26% versus 10%), though there are no differences in other measures of education. This is not surprising given Mongolia's overall high levels of primary and secondary schooling. Perceived project-driven improvements in the role of women were substantial, with 51% of member households, compared to 26% of non-member households, saying the role of women in the community has improved since the project's inception. Male and female respondents did not differ in this respect. A majority of member households attributed the improvement to the Community Organizations, while the main causes for the improvements mentioned by non-members were women's own initiatives and government action. Nearly all participants in the focus group discussions agreed that women have benefited from project training, have improved their skills, and have become more active in making products together. Many of the project's trainings were related to tasks that are usually performed by women in the household, such as processing milk and wool, leading to increases in social interactions, through meetings, workshops and trainings, and in economic contributions to the household.

When asked about the largest impact of the project, most survey respondents answered that the project brought people back together. Many referred to the sudden collapse of communism in 1990, after which the country was left disrupted and herders lost the ability to coordinate land use. The community project provided a venue for households to interact and work together. The project-funded information centers for each Community Organization facilitated this process, as herders met there and used the space for advertising, children's daycare, competitions and other activities.

## Discussion

Ecologically, the grazing management practices engendered by the project, especially member households' coordinated seasonal moves, appear to have had a beneficial effect on pasture condition. Community Organization-managed pasture areas, on average, had a longer growing season and higher peak plant growth than the non-member control sites. The long-term analysis showed that overall plant growth in the 1980 s and 1990 s prior to the conservation initiative was almost identical in both Community Organization and non-member sites, ruling out inherent differences between them as a cause for this finding. The long-term analysis also showed that, after the conservation program started, there was more forage available on Community Organization pastures than on non-member pastures during drought years in the Gobi.

Socioeconomically, Community Organization members were better off than comparable non-members in their districts, with 12% greater median annual income, a more diversified range of income-generating activities, better access to credit, and more household members attending university.

To better understand the factors that contributed to the project's achievements, in the focus group discussions and key informant interviews, the research team sought to identify the key success factors. Four were most frequently mentioned.

First, many project activities were community-driven. After some training, the project leaders provided the opportunity for herders to propose project activities and request funding from project funds. Proposals were evaluated based on their contributions to the project objectives of conservation and sustainable livelihoods. The Community Organizations also had to provide contributions themselves for the activities they wanted to undertake. As mentioned by one community member, “they didn't just give us things; they taught us to organize ourselves and achieve things together.” The result was greater local ownership of project activities. A strong indication of this local ownership is that some community organizers are still active several years after the project ended, even without pay. The benefits of community-driven development are well known within international development [27], [28] but perhaps less so within international conservation.

Second, the project facilitated knowledge exchange among herders by training local trainers to teach courses and organized workshops in which successful Community Organizations shared their approaches and knowledge with other herders. Moreover, the project sent community members abroad to participate in international conferences and events. Local people said they had learned a lot from these knowledge exchanges, and several mentioned that the exchanges helped instill pride in their accomplishments. Establishing peer-learning networks is an approach that has promise for conservation initiatives and echoes calls by others for learning networks in conservation [29], [30].

Third, the commitment of the project team to be present in the field was a crucial factor of success. Local people said that when the project started, they saw the project as foreign and were worried that the foreigners had come to take away their land. The project's structure, in which there was a local community organizer in each district who regularly visited each Community Organization, helped change this attitude. Because the project team resided close to the herders and worked with them on a local level, focus group participants said this made the herders more closely connected to the project team and motivated them. Perhaps it is because it is intuitively obvious, but there is little in the conservation or development literature that highlights the importance of having a day-to-day presence on the ground as a critical element for generating project participation and support. This is a clear benefit of the project structure that is worth noting for other conservation initiatives.

Fourth, the qualities of the community members and leader played an important role as a driver of success. A number of those interviewed noted that community members had to be willing to work hard and show initiative. Many herders said that those who are no longer active did not have the motivation to improve their lives and were not committed to work together with others. The Community Organization leader also had to be active and skilled. The project trained these leaders in management and communications, but participants said that the leaders also needed organizational capacity and negotiating skills to look after the interests of all community members. This supports the findings by others that strong local leadership is a crucial success factor in local resource management initiatives [31]–[33].

While the above success factors were important in creating conservation and livelihood benefits, the project's perceived premature ending may have reduced the long-term positive effects. Local people felt the project had ended just as it was starting to have a large impact. In the project's design, local government was supposed to take up the support for the Community Organizations, but because of elections, many of the newly elected officials were not knowledgeable about the Community Organizations and did not support them.

In the four years since the project ended, the area was hit by two consecutive years of drought, and an especially harsh winter in 2009–2010, which overwhelmed the capacity of pasture management strategies to conserve grassland. During the 2009–2010 winter, many households lost most or even all of their livestock, and many people migrated to urban areas such as Ulaanbaatar to look for work. The people who stayed behind also lost many animals, and the animals that did survive were less productive. Community Organization members, therefore, had less reason to come together and process products. Even with healthier pastures, better access to credit, and greater income levels, as was heard several times during the field work, the magnitude of the situation was too immense to be dealt with by Community Organizations. Mongolia has been impacted by climate change already [34], suggesting that the resilience of even a well-designed and implemented local conservation project is insufficient to meet the challenges of climate change in Mongolia.

## Supporting Information

Text S1 **Household survey.** The English version of the questionnaire used in the household survey.
(DOCX)

Data Set S1 **Data from household survey.** The data from the Gobi household survey in SPSS 15.0 format.
(SAV)
