# Supplementary material for: Measuring the Impacts of Community-based Grasslands Management in Mongolia's Gobi
Source: PLoS One. 2012 Feb 1;7(2):e30991. doi: 10.1371/journal.pone.0030991 (PMC3270020; doi:10.1371/journal.pone.0030991)
Supplement: Text S1 — Household survey. The English version of the questionnaire used in the household survey. (DOCX) [file pone.0030991.s001.docx]

**Household Survey Questionnaire**

**<FILL OUT THE DATA BELOW BEFORE THE INTERVIEW OR DIRECTLY AFTERWARDS!>**

| Name of the interviewer |  | **Household ID code** |  | |
| --- | --- | --- | --- | --- |
| Date of the interview |  | Start time of the interview |  | |
| Location of the interview (bag) |  | Finish time of the interview |  | |
| Local area name |  | Coordinates (lat/long) |  |  |
| Herder community name |  |  |  | |

| **INTERVIEWER INSTRUCTIONS**  **<INTERVIEW ONLY HOUSEHOLD MEMBERS WHO LIVE IN THE HOUSEHOLD >**  **<INTERVIEW ONLY ADULT HOUSEHOLD MEMBERS (18 YEARS AND OLDER)>**  **<INTRODUCE YOURSELF :>**  This is an independent research project from the Institute for Environmental Studies from the Netherlands to investigate the livelihoods and changes in the livelihoods of the people who live in and around the Gobi desert. The goal of the project is purely research; to better understand the issues that affect your daily life so other people in other countries can learn from your experiences.  I’m going to ask you a number of questions related to yourself and your household. Please note that any information you give me will be treated completely confidentially and not shared with anyone else. The information will only be used to characterize the area in which you live. All individual information will be added together to determine the average for the whole community, so nobody will be able to identify individual participants.  The interview will last about 1 hour.  Please answer as truthfully as possible. There are no right or wrong answers. |
| --- |

**1. HOUSEHOLD SITUATION, ACCESS TO SERVICES, AND ASSETS**

Please help me to make a complete list of the people who normally live and eat their meals together in this household, starting with the household head, then the immediate family and then the extended family. Please also count children who are living in school dormitories or with relatives, but for whom you pay the living and school expenses.

| I  D  C  O  D  E | 1.  NAME | 2.  SEX | 3.  How old is [NAME]? | 4.  What is the relationship of [NAME] to the household? | 5.  Is [NAME] attending school now? | 6.  What is the highest completed level of education by [NAME]? |
| --- | --- | --- | --- | --- | --- | --- |
|  | **<MARK THE ID-CODE OF THE RESPONDENT >** | MALE…...0 FEMALE .1 | YEARS | **<USE RELATIONSHIP CODING FROM BOX 1 BELOW>** | NO... 0  YES...1 | **<USE CODING FROM BOX BELOW>** |
| 1 |  |  |  |  |  |  |
| 2 |  |  |  |  |  |  |
| 3 |  |  |  |  |  |  |
| 4 |  |  |  |  |  |  |
| 5 |  |  |  |  |  |  |
| 6 |  |  |  |  |  |  |
| 7 |  |  |  |  |  |  |
| 8 |  |  |  |  |  |  |
| 9 |  |  |  |  |  |  |

| BOX 1: RELATIONSHIP CODES | |  | BOX 2: CODING HIGHEST EDUCATION LEVEL | |
| --- | --- | --- | --- | --- |
| HEAD OF THE HOUSEHOLD…………………………..……………. | 1 |  | NO SCHOOLING……………………….. | 1 |
| THE HUSBAND/WIFE OF THE HEAD OF THE HOUSEHOLD….. | 2 |  | PRIMARY ……………………………….. | 2 |
| SON/DAUGHTER OF THE HEAD OF THE HOUSEHOLD……….. | 3 |  | LOWER SECONDARY 8TH GRADE…. | 3 |
| BROTHER/SISTER OF THE HEAD OF THE HOUSEHOLD……... | 4 |  | VOCATIONAL……………………………. | 4 |
| FATHER/MOTHER OF THE HEAD OF THE HOUSEHOLD……… | 5 |  | UPPER SECONDARY 10TH GRADE… | 5 |
| OTHER (SPECIFY)……………………………………………………. | 6 |  | DIPLOMA………………………………… | 6 |
|  |  |  | UNIVERSITY…………………………….. | 7 |

1. Do all children between the age of 6 and 15 in your household attend school? If not, why not?
2. Yes, they all go to school
3. No, because we cannot afford the school expenses (stationary, clothes, dormitory/boarding)
4. No, because we need the children to help with work
5. No, because the school is too far away
6. No, other reason (specify)…………………………………………………………………
7. Could you tell me where you go for medical help if one of your household members has a serious medical problem?
8. Bag doctor
9. Soum centre
10. Aimag centre
11. Ulaanbaatar
12. Hoh hot
13. Other (specify)…………………………………………………………………
14. Thinking back to [before your household joined the community organization] **<OR IF THE HOUSEHOLD IS NOT PART OF A COMMUNITY, SAY: >** [2002, the year after the 1999-2001 dzuds] has the access of your household to medical services changed compared to the situation then? .
15. Worse
16. Same **>> SKIP NEXT QUESTION**
17. Better
18. If it changed, why did it change?

………………………………………………………………………………………………………………

………………………………………………………………………………………………………………

1. What are the first and second most important sources of energy/fuel used in your household for cooking and heating? **<TICK ONLY ONE OPTION IN EACH COLUMN>**

|  | 1^st^ most important | 2^nd^ most important |
| --- | --- | --- |
| 0 Animal dung |  |  |
| 1 Firewood |  |  |
| 2 Coal |  |  |
| 3 Briquettes (compressed fuel) |  |  |
| 4 Solar electricity |  |  |
| 5 Wind generated electricity |  |  |
| 6 Grid electricity |  |  |
| 7 Gas |  |  |
| 8 Other (specify) |  |  |

1. Can you tell me if your household owns any of the following items, and if so how many?

|  | Number |
| --- | --- |
| Ger |  |
| Cell phone |  |
| Radio |  |
| Motorcycle |  |
| Solar panel |  |
| Satellite dish and television |  |
| Car/truck |  |
| House/apartment in soum or aimag centre |  |

1. Does your household have access to electricity from the grid?
2. No
3. Yes

**2. LIVESTOCK, AND PASTURE MANAGEMENT**

1. Does your household currently own any livestock, or did you own any livestock last year?
2. No **>> SKIP TO Q46**
3. yes
4. If yes, could you specify how much livestock your household owns today and owned one year ago?

|  | Number today | Number one year ago |
| --- | --- | --- |
| Cows/yaks |  |  |
| Horses |  |  |
| Sheep |  |  |
| Goats |  |  |
| Camels |  |  |

1. Did your household buy any breeding stock last year?
2. No **>> SKIP NEXT QUESTION**
3. Yes
4. How many animals did your household buy for breeding during the last 12 months, and how much did you pay?

|  | Number | Price/animal |
| --- | --- | --- |
| Cows/yaks |  |  |
| Horses |  |  |
| Sheep |  |  |
| Goats |  |  |
| Camels |  |  |

1. During the last twelve months, how often did your household seek out veterinary services for your animals?

…………….times **>> IF NEVER, SKIP TO Q21**

1. What kind of assistance did you seek? **<MORE THAN ONE ANSWER POSSIBLE>**
2. Vaccination
3. Disease
4. Insemination
5. Other, please specify………………………………………..
6. Could you give an estimate of how much you had to pay for the veterinary services you used during the last twelve months?

…………….Tugrik **>> NOW SKIP TO Q22**

1. Why didn’t you seek out any veterinary services during the last 12 months?
2. No need
3. Couldn’t afford
4. Not available
5. Other, please specify…………………………………………………………………………….
6. Thinking back to [before your household joined the community organization] **<OR IF THE HOUSEHOLD IS NOT PART OF A COMMUNITY, SAY: >** [2002, the year after the 1999-2001 dzuds], did your household’s access to veterinary services change compared to the situation then?
7. Worse
8. Same **>> SKIP NEXT QUESTION**
9. Better
10. If your households access to veterinary services changed, why did it change?

………………………………………………………………………………………………………………

………………………………………………………………………………………………………………

1. Thinking back to [before your household joined the community organization] **<OR IF THE HOUSEHOLD IS NOT PART OF A COMMUNITY, SAY: >** [2002, the year after the 1999-2001 dzuds] , does your household now make more, less or the same seasonal moves in a year than compared to the situation then?
2. less
3. same **>> SKIP NEXT QUESTION**
4. more
5. If less or more, can you explain why?

………………………………………………………………………………………………………………

………………………………………………………………………………………………………………

1. Does your household own a winter camp, and if so, what kind of shelter does it have?
2. No **>> SKIP TO Q29**
3. Yes, three walls and roof
4. Yes, one wall, no roof
5. Other, please specify……………………………………………………….
6. Does your household have a certificate for the winter camp?
7. No
8. Yes

1. How long have you owned the winter camp? Since:

………….. **<IF RESPONDENT SAYS SINCE X YEARS AGO, CALCULATE WHICH YEAR THIS WAS AND CONFIRM >**

1. Is your household able to reserve winter pasture?
2. No
3. Yes **>> SKIP NEXT QUESTION**
4. If not, can you explain why not?

………………………………………………………………………………………………………………………….

1. Has this changed compared to the situation [“before your household joined the community organization”] / [“2002, the year after the 1999-2001 dzuds”]? **<SELECT ONE OF THE OPTIONS>**
2. More difficult now
3. No change **>> SKIP NEXT QUESTION**
4. Easier now
5. Why did it change?
6. Government action
7. Actions of organized communities
8. Climate change
9. Other, please specify…………………………………………………………………………………
10. Does your household have access to fenced off spring pasture?
11. No **>> SKIP NEXT QUESTION**
12. Yes
13. How long have you had access to fenced off spring pasture?

………….. **<IF RESPONDENT SAYS SINCE X YEARS AGO, CALCULATE WHICH YEAR THIS WAS AND CONFIRM >**

1. Was your household able to do otor last year, and if not, why not?
2. Yes
3. No, no reserved land
4. No, no transportation
5. No, other reason, please specify…………………………………………………………………………….
6. Is it easier or harder today to do otor compared to the situation [“before your household joined the community organization”] / [“2002, the year after the 1999-2001 dzuds”]? **<SELECT ONE OF THE OPTIONS>**
7. More difficult now
8. No change **>> SKIP NEXT QUESTION**
9. Easier now
10. Why did it change?
11. Government action
12. Actions of organized communities
13. Climate change
14. Other, please specify…………………………………………………………………………………
15. Has the average distance you need to take your livestock to water changed compared to the situation [“before your household joined the community organization”] / [“around 2002, the year after the 1999-2001 dzuds”]? **<SELECT ONE OF THE OPTIONS>**
16. Have to move further or more often than before
17. No change **>> SKIP NEXT QUESTION**
18. Don’t have to move so far or often as before
19. Why did this change?

………………………………………………………………………………………………………………………

1. How much hay and/or fodder did you grow last year for your household? How much hay and or fodder did you buy last year for your household? If you had to buy, how much did you pay for it? **<IF DONE TOGETHER WITH THE COMMUNITY ORGANIZATION, ASK FOR SHARE AND FILL OUT THAT NUMBER. IF NOTHING WAS GROWN OR BOUGHT FILL ZERO>**

|  | Amount grown/ prepared | | Amount bought | | Price/Unit | |
| --- | --- | --- | --- | --- | --- | --- |
|  | AMOUNT | UNIT | AMOUNT | UNIT | PRICE | UNIT |
| Hay |  |  |  |  |  |  |
| Fodder |  |  |  |  |  |  |

1. Was this amount of hay and fodder sufficient?
2. No
3. Yes **>> SKIP TO Q44**
4. If you didn’t grow (enough) hay or fodder, why not?

………………………………………………………………………………………………………………

………………………………………………………………………………………………………………

1. If you didn’t purchase (enough) hay or fodder, why not?

………………………………………………………………………………………………………………

………………………………………………………………………………………………………………

1. Do you feel your household is now better able to deal with dzud and drought than [“before your household joined the community organization”] / [“around 2002, the year after the 1999-2001 dzuds”]? **<SELECT ONE OF THE OPTIONS>**
2. No **>> SKIP NEXT QUESTION.**
3. Yes
4. Why has this changed?

………………………………………………………………………………………………………………

………………………………………………………………………………………………………………

1. Did you grow any crops or vegetables in 2009 for household consumption or to sell?
2. No **>> SKIP NEXT QUESTION.**
3. Yes
4. If yes, could you specify which crops or vegetables you grew?

|  | Amount grown in 2009 | |
| --- | --- | --- |
|  | Amount | Unit |
| Potatoes |  |  |
| Carrot |  |  |
| Onion |  |  |
| Cabbages |  |  |
| Other (specify)……… |  |  |
| Other (specify)……… |  |  |

1. Does your household now grow more or less crops or vegetables compared to the situation [“before your household joined the community organization”] / [“around 2002, the year after the 1999-2001 dzuds”]? **<SELECT ONE OF THE OPTIONS>**
2. Less
3. No change **>> SKIP NEXT QUESTION**
4. More
5. Why did it change?
6. Government action
7. Actions of organized communities
8. Other, please specify…

**3. PROFESSION, INCOME AND CREDIT**

I would now like to ask you about the income sources of the household.

Please note that all the information you give me will be treated completely confidentially and will be combined with all the other household data in the community to determine an average!

**< FIRST LIST ALL INCOME SOURCES. THEN CONTINUE WITH THE OTHER QUESTIONS PER SOURCE. IF THE RESPONDENT IS NOT SURE OR DOES NOT KNOW THE INCOME GENERATED BY OTHER HOUSEHOLD MEMBERS, PLEASE ASK HIM/HER TO TAKE A MOMENT TO CONSULT OTHER AVAILABLE FAMILY MEMBERS>**

| 49.  Could you indicate which sources of income your household had in 2009? |  | 50.  How much income did you have in 2009 from the following sources? | 51.  Where did you make your sales? | | 52.  Is this more or less com-pared to the situation [“before your household joined the community organization”] / [“around 2002, the year after the 1999-2001 dzuds”]?  **<SELECT ONE OF THE OPTIONS>** | | 53.  Who in the household is primarily responsible for this? |
| --- | --- | --- | --- | --- | --- | --- | --- |
|  |  |  | FAMILY & FRIENDS...  OTHER HERDERS….  VISITING TRADERS...  SOUM CENTRE……..  AIMAG CENTR….……  ULAANBAATAR……..  ACROSS BORDER (CHINA)……………… OTHER(SPECIFY)….. | 1  2  3  4  5  6  7  8 |  |  |  |
|  |  |  |  |  |  |  | **<USE “ID CODE FROM FIRST COLUMN ON PAGE 2. IF MORE THAN ONE PERSON, PUT ALL>** |
|  |  |  |  |  | LESS.............  SAME.………  MORE……… | 0  1  2 |  |
| **<TICK BOX IF INCOME SOURCE APPLIES>** |  | TUGRIK |  |  |  |  |  |
| *Selling live animals* |  |  |  | |  | |  |
| Cows/yaks |  |  |  | |  | |  |
| Horses |  |  |  | |  | |  |
| Sheep |  |  |  | |  | |  |
| Goats |  |  |  | |  | |  |
| Camels |  |  |  | |  | |  |
| *Selling unprocessed animal products* |  |  |  | |  | |  |
| Selling meat |  |  |  | |  | |  |
| Selling milk |  |  |  | |  | |  |
| Selling skins |  |  |  | |  | |  |
| Selling cashmere |  |  |  | |  | |  |
| Selling wool |  |  |  | |  | |  |
| Other……………….. |  |  |  | |  | |  |
| *Selling processed animal or other products* |  |  |  | |  | |  |
| Dairy products. |  |  |  | |  | |  |
| Leather products |  |  |  | |  | |  |
| Handicrafts |  |  |  | |  | |  |
| Briquettes |  |  |  | |  | |  |
| Other………………... |  |  |  | |  | |  |
|  |  |  |  | |  | |  |
| Selling vegetables |  |  |  | |  | |  |
| Ninja mining |  |  |  | |  | |  |
| Tourism (such as acting as a guide, renting of horses/ camels, providing housing and food) |  |  |  | |  | |  |
| Job (please specify the type of job, and give info per job)  a__________  b__________  c__________ |  |  |  | |  | |  |
| Government grants/pensions, etc. |  |  |  | |  | |  |
| Other (please specify type of source, and give information per source)  a__________  b__________  c__________ |  |  |  | |  | |  |

1. Do you feel you get a fair price for the animals or products that you sell?
2. No
3. Yes **>> SKIP NEXT QUESTION**

1. If not, can you indicate the two most important reasons why not?

|  | 1^st^ most important | 2^nd^ most important |
| --- | --- | --- |
| 0 I don’t have good price information |  |  |
| 1 I do not have transport to bring my products to good markets |  |  |
| 2 Traders have all the power |  |  |
| 3 There are cheaper products from abroad |  |  |
| 4 When we have to sell, prices are low |  |  |
| 5 Other, please specify |  |  |
| 6 Other, please specify |  |  |

1. Does your household now get better or worse prices than compared to the situation [“before your household joined the community organization”] / [“around 2002, the year after the 1999-2001 dzuds”]? **<SELECT ONE OF THE OPTIONS>**
2. Worse
3. Same **>> SKIP NEXT QUESTION**
4. Better
5. Why did this change?

………………………………………………………………………………………………………………

………………………………………………………………………………………………………………

1. How did the overall income situation of your household change compared to the situation [“before your household joined the community organization”] / [“around 2002, the year after the 1999-2001 dzuds”]? **<SELECT ONE OF THE OPTIONS>** Has it become harder or easier to meet the expenses that the household has?
2. Much harder
3. Harder
4. No change **>> SKIP NEXT QUESTION**
5. Easier
6. Much easier
7. Why did this change?

………………………………………………………………………………………………………………

………………………………………………………………………………………………………………

1. How many animals did you slaughter for household food last year?

|  | Number |
| --- | --- |
| Cows/yaks |  |
| Horses |  |
| Sheep |  |
| Goats |  |
| Camels |  |

1. During the last twelve months, how many times did (anyone in) your household borrow money from friends, family, traders, the buffer zone council, or any other person or institution?

…………….times **>> IF NEVER SKIP TO Q60**

1. Who did you or your household members borrow from (you can select more than one option)?
2. Family
3. Friends
4. Traders
5. Buffer zone council
6. Microcredit institution
7. Banks
8. Other, please specify……………………………………………………………………..

1. What did you use the loan(s) for?

…………….

……………. **>> NOW SKIP THE NEXT QUESTION**

1. If you or other household members didn’t borrow any money during the last 12 months, could you explain why not?
2. No need
3. You can only get a loan if you have collateral, and we don’t have
4. The process is too difficult
5. Repayment time is too short
6. Other, please specify…………………………………………………………………………………………..
7. Is it easier or harder today to get a loan than compared to the situation [“before your household joined the community organization”] / [“around 2002, the year after the 1999-2001 dzuds”]? **<SELECT ONE OF THE OPTIONS>**
8. Harder
9. No change **>> SKIP NEXT QUESTION**
10. Easier
11. If easier or harder, can you explain why?

………………………………………………………………………………………………………………

………………………………………………………………………………………………………………

**4. GOVERNANCE, PARTICIPATION & COMMUNITY INTERACTION**

1. In your opinion, how has the quality of the following components of your environment changed compared to the situation [“before your household joined the community organization”] / [“around 2002, the year after the 1999-2001 dzuds”]? **<SELECT ONE OF THE OPTIONS>**

|  |  | 0.  DECREASED | 1.  REMAINED STABLE | 2.  INCREASED | 3.  DON’T KNOW |
| --- | --- | --- | --- | --- | --- |
| A | Pasture grass abundance |  |  |  |  |
| B | Variety of pasture plants |  |  |  |  |
| C | Occurrence of medicinal plants |  |  |  |  |
| D | Occurrence of wildlife |  |  |  |  |
| E | Level of natural water sources |  |  |  |  |

1. How in your opinion has the establishment of the Gobi Gurvansaikhan National Park affected the environment?
2. Negatively
3. No effect
4. Positively
5. How in your opinion has the establishment of the Gobi Gurvansaikhan National Park affected the livelihood of your household?
6. Negatively
7. No effect
8. Positively
9. How has the relationship between the local people and the Gobi Gurvansaikhan National Park administration, such as its rangers, changed compared to the situation [“before your household joined the community organization”] / [“around 2002, the year after the 1999-2001 dzuds”]? **<SELECT ONE OF THE OPTIONS>**
10. Worsened
11. No change **>> SKIP NEXT QUESTION**
12. Improved
13. If it changed, can you explain why?

………………………………………………………………………………………………………………

………………………………………………………………………………………………………………

1. Who does your household generally discuss pasture management issues with, such as whether to reserve pasture, where to move to, when to move? **<MORE THAN ONE ANSWER POSSIBLE >**
2. Nobody
3. Neighbors
4. Organized community group
5. Bag or soum government
6. Other, please specify………………………………………………………………………………….
7. Did this change compared to the situation [“before your household joined the community organization”] / [“around 2002, the year after the 1999-2001 dzuds”]? **<SELECT ONE OF THE OPTIONS>** Who did you discuss with before? **<MORE THAN ONE ANSWER POSSIBLE >**
8. No change
9. Nobody
10. Neighbors
11. Organized community group
12. Bag or soum government
13. Other, please specify………………………………………………………………………………….
14. How often do disputes about the use of pasture land occur in this area?
15. Never **>> SKIP TO Q78**
16. Rarely
17. Occasionally
18. Regularly
19. If disputes occur, are they normally with herders from within the area or with people or groups from outside the area?
20. Local herders
21. Herders from outside
22. Don’t know
23. If a dispute occurs, how do you try to solve it?
24. Approach the soum or bag government
25. Negotiate with each other
26. Other, please specify…………………………………………………………………………………………..
27. Did the occurrence of disputes change compared to the situation [“before your household joined the community organization”] / [“around 2002, the year after the 1999-2001 dzuds”]? **<SELECT ONE OF THE OPTIONS>**
28. Decrease
29. No change
30. Increase
31. Do you feel that your household can influence the decisions of the local government that affect your lives?
32. No
33. Yes
34. Has this changed compared to the situation [“before your household joined the community organization”] / [“around 2002, the year after the 1999-2001 dzuds”]? **<SELECT ONE OF THE OPTIONS>**
35. Worsened
36. No change **>> SKIP NEXT QUESTION**
37. Improved
38. Why did this change?

………………………………………………………………………………………………………………

………………………………………………………………………………………………………………

1. Do you feel that support from the government has changed compared to the situation [“before your household joined the community organization”] / [“around 2002, the year after the 1999-2001 dzuds”]? **<SELECT ONE OF THE OPTIONS>**
2. Worsened
3. No change
4. Improved
5. Do you feel that the voice and opportunities of young people in the community has changed compared to the situation [“before your household joined the community organization”] / [“around 2002, the year after the 1999-2001 dzuds”]? **<SELECT ONE OF THE OPTIONS>**
6. Worsened
7. No change
8. Improved

**<ASK QUESTIONS 84 AND 85 ONLY IF THE RESPONDENT IS A WOMEN, OTHERWISE SKIP TO Q86>**

1. Are you satisfied by the role that women play in your community?
2. No
3. Yes
4. Has the role of women in your community changed compared to the situation [“before your household joined the community organization”] / [“around 2002, the year after the 1999-2001 dzuds”]? **<SELECT ONE OF THE OPTIONS>**
5. Worsened
6. No change
7. Improved
8. Is your household part of a herder group or herder community organization?
9. No **>> SKIP TO Q93**
10. Yes
11. What is the name of the community organization or group?

………………………………………….

1. In which year did your household join the herder group or community organization?

………….. **<IF RESPONDENT SAYS SINCE X YEARS AGO, CALCULATE WHICH YEAR THIS WAS AND CONFIRM>**

1. How was the community organization formed?
2. Bottom-up, without support
3. With support from the German Technical Assistance project
4. With support from the government
5. Other, please specify…………………………………………………………………………………….
6. Don’t know
7. Is any member of your household on the community organization council? If yes, who is?
8. No
9. Yes, namely …………………………….. **<PLEASE USE “ID CODE” FROM FIRST COLUMN ON PAGE 2>**
10. What kind of support has your household received through the community organization and the GTZ project?

|  |  |
| --- | --- |
| 0. Pasture land management training |  |
| 1. Training in briquette and stove production |  |
| 2. Training in wildlife monitoring |  |
| 3. Providing equipment for animal product processing |  |
| 4. Other, specify: |  |

1. Let me read you some statements on how community organization and the German Technical Cooperation project has influenced your household. For each statement, please tell me if you disagree, are neutral, agree or don’t know.

|  |  | 0.  Don’t know | 1.  Dis-agree | 2.  Neutral | 3.  Agree |
| --- | --- | --- | --- | --- | --- |
| A | The project has helped to increase my family’s income |  |  |  |  |
| B | Since the start of the project, we are less dependent on only livestock |  |  |  |  |
| C | The project helped to improve the position of women |  |  |  |  |
| D | The quality of the pastures has improved due to the project |  |  |  |  |
| E | The project has helped us to maintain our culture and tradition |  |  |  |  |
| F | Due to the project we feel safer in difficult times |  |  |  |  |
| G | The project has led to better access to markets to sell/buy our products |  |  |  |  |
| H | The project has improved cooperation between communities and government |  |  |  |  |
| I | The project has improved coordination of the movement of herds |  |  |  |  |
| K | The project has restricted the entry of outside herders to the communities pastures |  |  |  |  |

1. The interview is now competed. Do you have any questions for me?

………………………………………………………………………………………………………………

………………………………………………………………………………………………………………

Thank you very much for your time and interest in this study!
